# Supplementary material for: The efficiency in the ordinary hospital bed management: A comparative analysis in four European countries before the COVID-19 outbreak
Source: PLoS One. 2021 Mar 22;16(3):e0248867. doi: 10.1371/journal.pone.0248867 (PMC7984624; doi:10.1371/journal.pone.0248867)
Supplement: S1 Table — CMI = Case-Mix Index, PI = Performance Index, TOI = Turn-Over Interval, BOR = Bed Occupancy Rate, BTO = Bed Turn-Over, AvLOS = Average Length of Stay. Green cells specifies values that are within the relevant threshold: CMI > 1, PI < 1, 3<TOI<1, 75%<BOR<85%. (DOCX) [file pone.0248867.s001.docx]

**S1 Table. Cross-regional comparison of the results of bed management as well as complex and performance analysis. CMI = Case-Mix Index, PI = Performance Index, TOI = Turn-Over Interval, BOR = Bed Occupancy Rate, BTO = Bed Turn-Over, AvLOS = Average Length of Stay. Green cells specifies values that are within the relevant threshold: CMI > 1, PI < 1, 3<TOI<1, 75%<BOR<85%**

| **Nuts Id** | **Nuts Name** | **Complex and performance analysis** | | **Hospital Bed Management analysis** | | | | |
| --- | --- | --- | --- | --- | --- | --- | --- | --- |
|  |  | **CMI** | **PI** | **TOI** | | **BOR** | **BTO** | **AvLOS** |
| **France** | | | | | | | | |
| FR10 | Ile-de-France | 1,05 | 0,91 | 3,25 | | 74% | 29,46 | 9,14 |
| FRB0 | Centre - Val de Loire | 1,05 | 0,93 | 2,73 | | 77% | 30,58 | 9,21 |
| FRC1 | Bourgogne | 1,07 | 0,91 | 2,74 | | 77% | 30,84 | 9,10 |
| FRC2 | Franche-Comté | 1,06 | 0,92 | 2,48 | | 79% | 31,61 | 9,07 |
| FRD1 | Basse-Normandie | 1,06 | 0,87 | 2,74 | | 76% | 32,35 | 8,54 |
| FRD2 | Haute-Normandie | 1,05 | 0,87 | 2,35 | | 79% | 32,99 | 8,71 |
| FRE1 | Nord-Pas de Calais | 1,06 | 0,80 | 3,18 | | 71% | 33,68 | 7,65 |
| FRE2 | Picardie | 1,05 | 0,89 | 2,47 | | 78% | 33,14 | 8,55 |
| FRF1 | Alsace | 1,05 | 0,89 | 3,46 | | 71% | 30,38 | 8,56 |
| FRF2 | Champagne-Ardenne | 1,05 | 0,90 | 2,53 | | 77% | 32,84 | 8,59 |
| FRF3 | Lorraine | 1,05 | 0,88 | 3,30 | | 72% | 31,43 | 8,31 |
| FRG0 | Pays de la Loire | 1,06 | 0,83 | 2,76 | | 75% | 33,20 | 8,23 |
| FRH0 | Bretagne | 1,12 | 0,88 | 3,07 | | 75% | 30,27 | 8,99 |
| FRI1 | Aquitaine | 1,06 | 0,89 | 3,53 | | 71% | 29,69 | 8,76 |
| FRI2 | Limousin | 1,08 | 0,89 | 5,57 | | 61% | 25,51 | 8,74 |
| FRI3 | Poitou-Charentes | 1,05 | 0,84 | 2,79 | | 74% | 33,50 | 8,10 |
| FRJ1 | Languedoc-Roussillon | 1,07 | 0,90 | 3,91 | | 70% | 27,97 | 9,14 |
| FRJ2 | Midi-Pyrénées | 1,08 | 0,85 | 3,17 | | 73% | 30,54 | 8,78 |
| FRK1 | Auvergne | 1,08 | 0,97 | 3,69 | | 72% | 27,34 | 9,66 |
| FRK2 | Rhône-Alpes | 1,08 | 0,87 | 3,04 | | 74% | 30,99 | 8,74 |
| FRL0 | Alpes-Côte d’Azur | 1,05 | 0,95 | 3,11 | | 76% | 28,45 | 9,72 |
| FRM0 | Corse | 1,05 | 0,98 | 1,89 | | 84% | 30,54 | 10,06 |
| **Germany** | | | | | | | | |
| DE1 | Baden-Wurttemberg | 1,02 | 0,99 | 3,12 | | 75% | 29,71 | 9,16 |
| DE2 | Bayern | 1,01 | 0,97 | 3,05 | | 74% | 30,70 | 8,84 |
| DE3 | Berlin | 0,99 | 0,88 | 1,35 | | 85% | 39,79 | 7,82 |
| DE4 | Brandenburg | 1,03 | 1,05 | 2,33 | | 81% | 29,80 | 9,92 |
| DE5 | Bremen | 0,98 | 0,84 | 2,04 | | 78% | 38,49 | 7,44 |
| DE6 | Hamburg | 0,99 | 0,89 | 1,55 | | 84% | 38,42 | 7,95 |
| DE7 | Hessen | 1,02 | 1,03 | 2,84 | | 77% | 29,38 | 9,58 |
| DE8 | Mecklenburg-Vorpommern | 1,08 | 1,15 | 3,79 | | 74% | 25,10 | 10,75 |
| DE9 | Niedersachsen | 1,01 | 0,97 | 2,69 | | 77% | 31,49 | 8,90 |
| DEA | Nordrhein-Westfalen | 0,99 | 0,92 | 2,39 | | 78% | 34,31 | 8,24 |
| DEB | Rhineland-Pfalz | 1,01 | 0,97 | 2,76 | | 76% | 31,51 | 8,83 |
| DEC | Saarland | 1,01 | 1,02 | 2,06 | | 82% | 31,82 | 9,41 |
| DED | Sachsen | 0,99 | 1,00 | 2,76 | | 77% | 30,81 | 9,09 |
| DEE | Sachsen-Anhalt | 0,99 | 0,99 | 2,52 | | 77% | 32,65 | 8,66 |
| DEF | Schleswig-Holstein | 1,07 | 1,15 | 3,29 | | 76% | 26,70 | 10,38 |
| DEG | Thüringen | 1,01 | 1,04 | 2,82 | | 77% | 29,53 | 9,54 |
| **Italy** | | | | | | | | |
| ITC1 | Piemonte | 0,96 | 1,08 | 2,56 | 77% | | 32,19 | 8,78 |
| ITC2 | Valle d'Aosta | 0,95 | 0,93 | 2,47 | 76% | | 35,00 | 7,96 |
| ITC3 | Liguria | 0,96 | 0,96 | 1,66 | 83% | | 36,51 | 8,34 |
| ITC4 | Lombardia | 0,96 | 0,97 | 2,15 | 79% | | 35,53 | 8,13 |
| ITF1 | Abruzzo | 0,92 | 0,90 | 1,96 | 80% | | 37,83 | 7,69 |
| ITF2 | Molise | 0,93 | 0,91 | 2,09 | 79% | | 36,58 | 7,88 |
| ITF3 | Campania | 0,91 | 0,91 | 2,27 | 75% | | 39,62 | 6,94 |
| ITF4 | Puglia | 0,90 | 0,86 | 1,87 | 79% | | 40,30 | 7,18 |
| ITF5 | Basilicata | 0,93 | 0,94 | 2,67 | 74% | | 35,01 | 7,76 |
| ITF6 | Calabria | 0,92 | 0,90 | 3,23 | 70% | | 34,15 | 7,46 |
| ITG1 | Sicilia | 0,94 | 0,94 | 2,30 | 77% | | 36,07 | 7,82 |
| ITG2 | Sardegna | 0,92 | 0,83 | 3,03 | 70% | | 36,03 | 7,10 |
| ITH1 | Provincia Autonoma di Bolzano | 0,97 | 0,93 | 2,03 | 79% | | 37,22 | 7,77 |
| ITH2 | Provincia Autonoma di Trento | 0,96 | 1,11 | 2,24 | 81% | | 31,32 | 9,41 |
| ITH3 | Veneto | 0,96 | 1,02 | 2,05 | 80% | | 34,92 | 8,40 |
| ITH4 | Friuli-Venezia Giulia | 0,92 | 0,88 | 2,55 | 75% | | 36,34 | 7,50 |
| ITH5 | Emilia-Romagna | 0,96 | 0,89 | 2,10 | 78% | | 37,96 | 7,52 |
| ITI1 | Toscana | 0,94 | 0,81 | 2,14 | 76% | | 40,95 | 6,78 |
| ITI2 | Umbria | 0,92 | 0,84 | 1,35 | 84% | | 42,96 | 7,14 |
| ITI3 | Marche | 0,93 | 0,97 | 1,96 | 80% | | 36,55 | 8,02 |
| ITI4 | Lazio | 0,92 | 0,99 | 3,21 | 72% | | 31,82 | 8,26 |
| **Spain** | | | | | | | | |
| ES11 | Galicia | 0,89 | 0,97 | 3,48 | | 69% | 32,60 | 7,71 |
| ES12 | Principado de Asturias | 0,91 | 0,93 | 3,00 | | 72% | 34,19 | 7,68 |
| ES13 | Cantabria | 0,88 | 1,25 | 4,06 | | 67% | 29,91 | 8,15 |
| ES21 | País Vasco | 0,91 | 1,26 | 2,19 | | 80% | 33,91 | 8,57 |
| ES22 | Comunidad Foral de Navarra | 0,91 | 1,14 | 3,52 | | 69% | 32,28 | 7,79 |
| ES23 | La Rioja | 0,87 | 1,19 | 2,66 | | 75% | 33,68 | 8,18 |
| ES24 | Aragón | 0,89 | 1,02 | 4,04 | | 65% | 31,28 | 7,63 |
| ES30 | Comunidad de Madrid | 0,89 | 0,93 | 2,38 | | 74% | 39,52 | 6,86 |
| ES41 | Castilla y León | 0,90 | 1,20 | 3,13 | | 73% | 31,39 | 8,50 |
| ES42 | Castilla-La Mancha | 0,88 | 0,90 | 2,93 | | 71% | 35,97 | 7,22 |
| ES43 | Extremadura | 0,88 | 0,98 | 3,95 | | 65% | 32,11 | 7,41 |
| ES51 | Cataluña | 0,91 | 1,09 | 3,13 | | 73% | 31,02 | 8,64 |
| ES52 | Comunidad Valenciana | 0,88 | 0,76 | 2,49 | | 70% | 43,22 | 5,95 |
| ES53 | Illes Balears | 0,89 | 0,88 | 2,62 | | 72% | 38,79 | 6,79 |
| ES61 | Andalucía | 0,86 | 0,86 | 3,20 | | 67% | 37,42 | 6,55 |
| ES62 | Región de Murcia | 0,87 | 0,94 | 3,99 | | 65% | 32,21 | 7,34 |
| ES63 | C. A. de Ceuta | 0,79 | 0,71 | 4,78 | | 53% | 35,84 | 5,40 |
| ES64 | C. A. de Melilla | 0,73 | 0,92 | 1,92 | | 75% | 46,87 | 5,87 |
| ES70 | Canarias | 0,88 | 1,14 | 4,12 | | 68% | 28,00 | 8,91 |
